# Phenolic Enriched Extract of *Baccharis trimera* Presents Anti-inflammatory and Antioxidant Activities

**DOI:** 10.3390/molecules17011113

**Published:** 2012-01-23

**Authors:** Cristiane B. de Oliveira, Lucimara N. Comunello, Adroaldo Lunardelli, Robson H. Amaral, Melissa G. S. Pires, Gabriela Lucas da Silva, Vanusa Manfredini, Carmen Regla Vargas, Simone C. B. Gnoatto, Jarbas R. de Oliveira, Grace Gosmann

**Affiliations:** 1 Faculdade de Farmácia, Universidade Federal do Rio Grande do Sul (UFRGS), Porto Alegre, RS 90610-000, Brazil; Email: cristiane_gabi@yahoo.com.br (C.B.O.); maracomunello@hotmail.com (L.N.C.); simone.gnoatto@ufrgs.br (S.C.B.G.); 2 Laboratório de Biofísica Celular e Inflamação, Pontifícia Universidade Católica do Rio Grande do Sul (PUCRS), Porto Alegre, RS 90619-900, Brazil; Email: adroaldo@via-rs.net (A.L.); robson.henrich@yahoo.com.br (R.H.A.); mgspires@pucrs.br (M.G.S.P.); gabi-ls@hotmail.com (G.L.S.); jarbas@pucrs.br (J.R.O.); 3 Hospital de Clínicas de Porto Alegre, Serviço de Genética Médica, UFRGS, Porto Alegre, RS 93050-150, Brazil; Email: vanusa_manfredini@yahoo.com.br (V.M.); crvargas@hcpa.ufrgs.br (C.R.V.)

**Keywords:** anti-inflammatory, antioxidant, *Baccharis trimera*, phenolic compounds, pleurisy, saponin, TAR

## Abstract

*Baccharis trimera* is a plant popularly used as a tea and to treat gastrointestinal diseases and inflammatory processes as well. The total phenolic content was determined and the antioxidant and anti-inflammatory activities of six extracts (dichloromethane, ethyl acetate, butanol, aqueous, saponin and phenolic) from *B. trimera* were evaluated. Using carrageenan-induced pleurisy as a model of acute inflammation, the phenolic extract at 15 mg/kg decreased significantly the analyzed parameters when compared to the carrageenan group (*p* < 0.05), thus showing potential anti-inflammatory activity. The total phenolic content and antioxidant activity were evaluated by the Folin-Ciocalteau and DPPH methods, respectively. Phenolic and ethyl acetate extracts presented higher antioxidant activity (*p* < 0.05) than ascorbic acid. The phenolic extract also showed the highest antioxidant potential in relation to the other extracts, thus suggesting that the antioxidant and anti-inflammatory activities were due to the presence of phenolic compounds.

## 1. Introduction

*Baccharis trimera*, a member of the Asteraceae, is a shrub native to South Brazil, Paraguay, Uruguay and Argentina. Medicinal teas prepared from its aerial parts are used in folk medicine to treat not only gastrointestinal and liver diseases, but also inflammatory processes [1] and itis the official species of the Brazilian Pharmacopoeia [2]. Literature mainly cites the presence of volatile oil, flavones, flavonol, saponins and diterpenes [3,4,5,6,7]. The flavonoids reported so far to *B. trimera* were apigenin, 7,4'-di-*O*-methyl-apigenin, cirsimaritin, eupatorin, genkwanin, hispidulin, isoquercetin, luteolin, nepetin, quercetin, 3-*O*-methylquercetin, 5,6-dihydroxy-7,3',4'-trimethoxyflavone and rutin [8,9,10]. In relation to its pharmacological activity, several studies demonstrated the anti-inflammatory activity of its ethanol and aqueous extracts, as well as analgesic, antioxidant, antihepatotoxic and antimutagenic activities [8,9,11,12]. Recently, our group demonstrated that the crude aqueous extract of *B. trimera* presented anti-inflammatory activity at the doses of 400 and 800 mg/kg [13].

Considering the previous biological activity studies of *B. trimera*,chemical and pharmacological studies using bioassay-guided fractionation of *B. trimera* were initiated by our group in order to identify its anti-inflammatory and antioxidant compounds. The investigation of its lead compounds should contribute to new drug discovery.

## 2. Results and Discussion

In this study, the phenolic content, the anti-inflammatory and antioxidant activities of *B*. *trimera* extracts were studied using a bioassay-guided fractionation. Extracts with major compounds were obtained as enriched phenolic and saponin extracts.

TLC analyses showed a predominance of phenolic and terpenoid compounds in the ethyl acetate and butanol fractions. This was verified by TLC using anisaldehyde-H_2_SO_4_/100 °C through visualization of violet spots in both fractions, a color characteristic of terpenoid compounds. Also, using the Natural Reagent A/UV_356_, it was possible to observe yellow spots corresponding to phenolic compounds present in these same *B. trimera* fractions. Both fractions were pooled together and submitted to molecular permeation chromatography to obtain one extract presenting only yellow spots, named the phenolic (PHE) extract, and another one presenting only violet spots, and named saponin (SAP) extract.

The phenolic content determined by the Folin-Ciocalteau method correlated with the antioxidant activity determined using the DPPH test. The total phenolic content of samples ranged from 107 to 1,482 mg of gallic acid/g of dry sample (Table 1). The phenolic (1,482.02 ± 50.41 mg of gallic acid/g of dry sample) and the ethyl acetate extracts (1,387.95 ± 42.45 mg of gallic acid/g of dry sample) presented the highest amounts of phenolic compounds.

**Table 1:** Total phenolic content in *B. trimera* extracts.

Samples	Phenolic content ^a^ (GAE/g)
Crude ethanol	261.59 ± 8.75
Dichloromethane	126.93 ± 5.52
Ethyl acetate	1387.95 ± 42.45
Butanol	107.71 ± 2.07
Aqueous	455.29 ± 24.9
Saponin	223.02 ± 28.86
Phenolic	1482.02 ± 50.41

^a^ Results are expressed as means ± S.D (n = 3) in mg of gallic acid equivalent/g of dry weight.

Moreover the phenolic extract presented the best antioxidant activity (IC_50_ 1.57 μg/mL) in comparison to ascorbic acid (AA) (IC_50_ 3.38 μg/mL) and samples (Table 2). As to other *Baccharis* species, the antioxidant activity IC_50_ values of crude extract, ethyl acetate and aqueous fraction from *B. pentlandii* DC. were 56.7 ± 1.2, 15.0 ± 0.3 and 50.3 ± 1.2 µg/mL, respectively, and from *B. platipoda* were 49.5 ± 0.8, 20.5 ± 1.3 and 73.3 ± 8.6 µg/mL, respectively [14]. Considering isolated compounds, quercetin (IC_50_ = 3.73 μg/mL) demonstrated very good antioxidant activities [15].

**Table 2:** IC_50_ and corresponding AEAC obtained using DPPH assay in *B. trimera* extracts.

Samples	IC_50_ (µg/mL)	AEAC (g)
Ascorbic acid (AA)	3.38	1
Crude ethanol	15.49	4.58
Dichloromethane	29.95	8.85
Ethyl acetate	6.25	1.85
Butanol	8.24	2.44
Aqueous	50.87	15.03
Saponin	10.25	3.03
Phenolic	1.57	0.46
Quercetin	2.41	0.71
Luteolin	2.84	0.84

AEAC, ascorbic acid equivalent antioxidant capacity.

**Figure 1:**
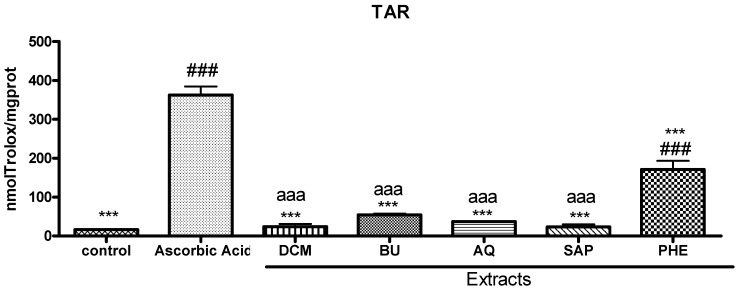
Total antioxidant reactivity (TAR, nmL/Trolox/mg protein) of extracts (DCM: 0.5 mg/mL; BU: 1 mg/mL; AQ: 1 mg/mL; SAP: 1 mg/mL and PHE: 1 mg/mL) and control. Results are expressed as means ± S.D. Significant differences determined by ANOVA complemented with Tukey’s test. ^###^*p* < 0.001 control *vs.* fractions and acid ascorbic, *** *p* < 0.001 acid ascorbic *vs.* control and extracts, ^aaa^*p* < 0.001 phenolic extract *vs.* other extracts (DCM, BU, AQ and SAP).

Regarding the TAR determination, ascorbic acid (AA) was used as a reference due to its potent antioxidant activity. The phenolic extract presented a TAR value comparable to AA (Figure 1) moreover this sample showed a significant increase (*p* < 0.001) in total antioxidant reactivity in comparison to the other samples tested and the control. 

Inflammation is a protective process that is essential for the preservation of the integrity of the organism in the events of chemical, physical and infectious damages. In our experiments, intrapleural injection of carrageenan induced an acute inflammatory reaction, characterized by marked accumulation of a volume of pleural exudates, plasma exudation and intense migration of PMNs in the pleural cavities. In previous phytochemical studies of some *Baccharis* species, the potential antioxidant activity of crude extract from *B. trimera* [12] was identified by our group. Also, our group determined that aqueous extract from *B. trimera* at 400 and 800 mg/kg presented anti-inflammatory activity using the carragenan pleurisy model i.p. [13].

Herein the *B. trimera* extracts assayed (DCM, EA, BU and AQ at 25, 50 and 75 mg/kg, i.p.) (Table 3) on pleurisy inflammation induced by carrageenan were effective in inhibiting the volume of exudation, leukocyte migration, leukocytes differential cell count and protein concentration.

**Figure 2:**
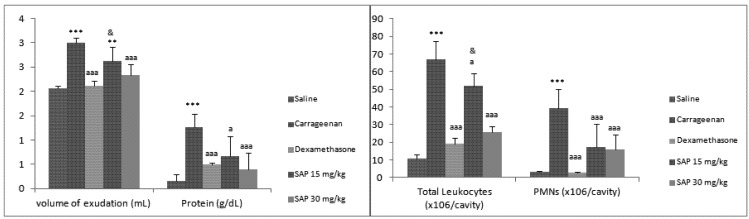
Effects of SAP *B. trimera* extract on inflammation induced by carrageenan. Significant differences were determined by ANOVA complemented with Bonferroni’s test. Results are expressed as means ± S.D (n = 7). * *p* < 0.05, ** *p* < 0.01, *** *p* < 0.001 *vs.* saline; ^a^*p* < 0.05, ^aa^*p* < 0.01, ^aaa^*p* < 0.001 *vs.* carrageenan and ^&^*p* < 0.05, ^&&^*p* < 0.01, ^&&&^*p* < 0.001 *vs.* dexamethasone.

Moreover, when testing the enriched extracts containing phenolic compounds at doses of 5 and 15 mg/kg or saponins at doses of 15, and 30 mg/kg, it could be demonstrated that the phenolic enriched extract presented the best anti-inflammatory activity at 15 mg/kg. So, animals treated with saponin extract (Figure 2) at doses of 15 and 30 mg/kg, i.p., had their volume of exudate significantly decreased at 30 mg/kg (22.34 ± 0.22 % inhibition, *p* < 0.01) when compared to carrageenan. In both doses, SAP considerably reduced the leukocyte migration (22.04 ± 6.99 and 61.96 ± 3.44% inhibition, *p* < 0.05), and the neutrophil decreased as well (56.34 ± 13.3 and 59.72 ± 8.51% inhibition, *p* < 0.001) when compared to carrageenan. Protein concentration significantly decreased at doses of 15 and 30 mg/kg, i.p. (47.04 ± 0.41 and 68.78 ± 0.35 % inhibition, *p* < 0.05) when compared to carrageenan.

**Table 3:** Effects of *B. trimera* extracts on pleurisy inflammation parameters induced by carrageenan and on nitric oxide levels.

Groups/dose	volume of exudation (mL)	Total Leukocytes (×10^6^/cavity)	PMNs (×10^6^/cavity)	Protein (g/dL)	NO (nmol/cavity)
Saline	2.06 ± 0.05	10.57 ± 2.55	3.12 ± 0.38	0.16 ± 0.12	4.44 ± 3.37
Carrageenan	2.96 ± 0.15 ***	37.47 ± 21.88 **	43.70 ± 18.71 ***	2.25 ± 0.46 ***	70.53 ± 18.06
Dexamethasone	2.12 ± 0.09 ^aaa^	19.05 ± 3.57	2.79 ± 0.46 ^aaa^	0.50 ± 0.04 ^aaa^	39.97 ± 11.73
DCM 25 mg/kg	2.46 ± 0.13 **^aaa&&^	8.79 ± 2.53 ^aa^	5.90 ± 0.42 ^aa^	0.54 ± 0.20 ^aaa^	12.67 ± 4.48 ^aaa&^
DCM 50 mg/kg	2.42 ± 0.19 **^aaa&^	7.52 ± 0.98 ^aa^	4.30 ± 0.49 ^aaa^	0.62 ± 0.34 ^aaa^	17.71 ± 4.17 ^aaa^
DCM 75 mg/kg	2.34 ± 0.18 ^aaa^	8.03 ± 1.99 ^aa^	4.00 ± 0.69 ^aaa^	0.69 ± 0.12 ^aaa^	19.93 ± 4.49 ^aaa^
EA 25 mg/kg	2.45 ± 0.23 **^aaa&&^	17.71 ± 7.50 ^aa^	10.45 ± 5.16 ^a^	0.48 ± 0.26 ^aaa^	17.51 ± 6.94 ^aaa^
EA 50 mg/kg	2.35 ± 0.12 *^aaa^	11.97 ± 4.04 ^aaa^	2.66 ± 1.96 ^aa^	0.59 ± 0.43 ^aaa^	17.69 ± 15.89 ^aaa^
EA 75 mg/kg	2.32 ± 0.08 ^aaa^	6.42 ± 1.34 ^aaa&^	2.43 ± 0.15 ^aa^	0.48 ±0.21 ^aaa^	20.62 ± 26.93 ^aaa^
BU 25 mg/kg	2.51 ± 0.23 ***^aaa&&&#^	20.20 ± 14.54 *^a^	15.25 ± 12.79 ^a^	1.31 ± 0.55 ***^a&^	15.91 ± 5.54 ^aaa&&^
BU 50 mg/kg	2.33 ± 0.13 ^aaa^	11.45 ± 3.00 ^aa^	2.86 ± 0.58 ^aaa^	1.01 ± 0.37 **^aa^	12.26 ± 4.44 ^aaa&&^
BU 75 mg/kg	2.25 ± 0.14 ^aaa^	9.93 ± 2.40 ^aaa^	2.80 ± 0.42 ^aaa^	0.92± 0.28 *^aaa^	10.67 ± 3.44 ^aaa&&^
AQ 25 mg/kg	2.35 ± 0.34 ^aaa^	15.12 ± 11.99 ^aa^	38.15 ± 4.59 *	0.99 ±0.81 *^aaa^	26.75 ± 17.00 ^aaa^
AQ 50 mg/kg	2.16 ± 0.15 ^aaa^	7.28 ± 6.40 ^aaa^	2.00 ± 0.28 ^aa^	0.44 ± 0.22 ^aaa^	25.97 ± 17.00 ^aaa^
AQ 75 mg/kg	2.16 ± 0.12 ^aaa^	8.96 ± 5.46 ^aaa^	3.70 ± 1.10 ^aa^	0.34 ± 0.26 ^aaa^	28.77 ± 24.49 ^aaa^

Results are expressed as means ± S.D (n = 7). * *p* < 0.05, ** *p* < 0.01, *** *p* < 0.001 *vs.* saline; ^a^*p* < 0.05, ^aa^*p* < 0.01, ^aaa^*p* < 0.001 *vs.* carrageenan and ^&^*p* < 0.05, ^&&^*p* <0.01, ^&&&^*p* < 0.001 *vs.* dexamethasone (ANOVA complemented with Bonferroni’s test).

**Figure 3:**
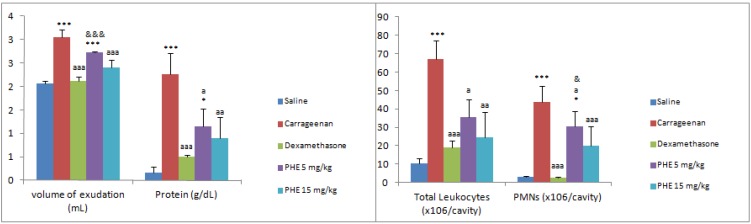
Effects of PHE *B. trimera* extract on inflammation induced by carrageenan. Significant differences were determined by ANOVA complemented with Bonferroni’s test. Results are expressed as means ± S.D (n = 7). * *p* < 0.05, ** *p* < 0.01, *** *p* < 0.001 *vs.* saline; ^a^*p* < 0.05, ^aa^*p* < 0.01, ^aaa^*p* < 0.001 *vs.* carrageenan and ^&^*p* < 0.05, ^&&^*p* < 0.01, ^&&&^*p* < 0.001 *vs.* dexamethasone.

Animals treated with phenolic extract (at doses of 5 and 15 mg/kg, i.p.) (Figure 3) had their volume of exudates decreased only at dose of 15 mg/kg, i.p. (21.31 ± 0.16% inhibition, *p* < 0.001) when compared to carrageenan and they also had their leukocyte migration significantly decreased at doses of 5 and 15 mg/kg, i.p. (46.79 ± 19.65 and 63.35 ± 13.53% inhibition, *p* < 0.05). The neutrophil influx also significantly decreased (30.00 ± 18.01 and 54.42 ± 11.78% inhibition, *p* < 0.05) and the reduction of protein concentration occurred from 49.38 ± 0.40 to 60.01 ± 0.45% of inhibition (*p* < 0.05), at the doses of 5 and 15 mg/kg, i.p. when compared to carrageenan.

**Figure 4:**
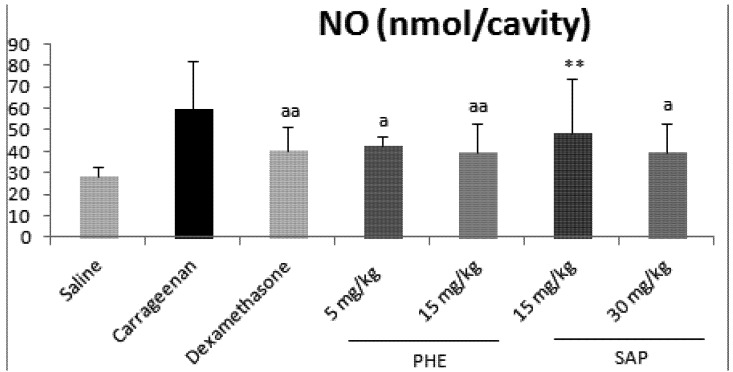
Effects of *B. trimera* extracts (PHE and SAP) on nitric oxide levels. Significant differences were determined by ANOVA complemented with Bonferroni’s test. Results are expressed as means ± S.D (n = 7). * *p* < 0.05, ** *p* < 0.01, *** *p* < 0.001 *vs.* saline; ^a^*p* < 0.05, ^aa^*p* < 0.01, ^aaa^*p* < 0.001 *vs.* carrageenan and ^&^*p* < 0.05, ^&&^*p* < 0.01, ^&&&^*p* < 0.001 *vs.* dexamethasone.

Nitric oxide (NO) is another important pro-inflammatory mediator involved in exudation and leukocyte migration in the inflammatory process. Evidence for the role of NO as a vasodilator and hyperpolarizing agent in the endothelial cells has been discussed [16]. Once again, *B. trimera* extracts demonstrated significant inhibition of NO levels. The extracts DCM, EA, BU and AQ at doses of 25, 50 and 75 mg/kg, i.p., presented percentages of inhibition varying from 59.21 to 77.44 (Table 3). Saponin extract only decreased the NO production at 30 mg/kg while phenolic extract at doses of 5 and 15 mg/kg, i.p. caused a significant decrease of NO production when compared to carrageenan (Figure 4). 

## 3. Experimental

### 3.1. Plant Material

*B. trimera* (Less.) DC plants were collected in Rio Grande do Sul, Brazil and identified by Prof. Dr. Nelson Matzenbacher from Pontifícia Universidade Católica do Rio Grande do Sul (PUCRS), Brazil. A voucher specimen is deposited at the Herbarium (ICN 152107) of the Universidade Federal do Rio Grande do Sul. Aerial parts were air-dried and powdered.

### 3.2. Chemical

All chemicals were purchased from Sigma (Milwaukee, WI, USA), Fluka Chemie (Buchs, Switzerland) and Merck (Darmstadt, Germany) and they were of analytical grade.

### 3.3. Extraction

Aerial parts of *B. trimera* (1 kg) were extracted by maceration with ethanol 96% (plant:solvent, 1:10, w/v, 2 × 15 days). The crude ethanol extract (CE, 130 g) was obtained after filtration and evaporation under vacuum. Aerial parts of *B. trimera* (50 g) were also exhaustively and successively extracted using a Soxhlet apparatus and the appropriate solvent, which provided in the corresponding dichloromethane (DCM, 10%), ethyl acetate (EA, 4%) and butanol (BU, 3%) extracts. The plant residue was submitted to decoction in order to obtain the residual aqueous extract (AQ, 14%). The ethyl acetate and butanol fractions were pooled together and submitted to molecular permeation chromatography on Sephadex LH-20 (Pharmacia) eluted with 99% ethanol to obtain the phenolic (PHE, 19%) and the saponin (SAP, 63%) extracts. The organic solvents were evaporated under reduced pressure to dryness and the aqueous extract was lyophilized. All extracts were chemically characterized according our previous results [5].

### 3.4. Chromatographic Analysis

Phytochemical profile of *B. trimera* was carried out by thin-layer chromatography (TLC) on silica gel plates GF_254_ using as mobile phase chloroform-ethanol-acetic acid (CHCl_3_:EtOH:HOAc, 60:40:6 v/v) and two color reagents: anisaldehyde-H_2_SO_4_ to detect terpenoid compounds and Natural Reagent A to visualize phenolic compounds [5,17].

### 3.5. Total Phenolic Determination

The total phenolic content was determined using the Folin-Ciocalteau method [18]. The standard curve was plotted using gallic acid (10–30 µg/mL) which regression equation was y = 0.0275x + 0.0104 (R^2^ = 0.9993). The absorbance was measured at 750 nm and the results were expressed as mg of gallic acid equivalents per gram of dry weight sample (GEA/g). The experiment was conducted in triplicate.

### 3.6. DPPH Assay

The scavenging effect on DPPH was determined according to Brand-Williams *et al*. [19] using sample and reference substances concentrations from 25 to 200 µg/mL. The absorbance was measured at 515 nm and the experiment was conducted in triplicate. The DPPH solution in methanol was used as a negative control. As a positive control, ascorbic acid (AA), quercetin and luteolin were used. The antioxidant activity (AOA) was expressed as the concentration (μg/mL) that inhibited DPPH radical formation by 50% (IC_50_) in comparison to the negative control. The lower the IC_50_ the higher the AOA. Results were also expressed as AEAC (ascorbic acid equivalent antioxidant capacity) in grams and calculated as follows:


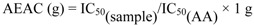


### 3.7. Total Antioxidant Reactivity (TAR)

TAR, which represents the quality of the antioxidant sample, was determined by measuring the luminal chemiluminescence intensity induced by 2,2'-azo-bis-(2-amidinopropane) (ABAP) according to Lissi *et al*. [20]. The background chemiluminescence was measured by adding 4 mL of 2 mM ABAP (in 0.1 M glycine buffer, pH 8.6) into a glass scintillation vial. Fifteen microliters of luminol (4 mM) was added to each vial and the chemiluminescence was measured (basal value). Ten microliters from 25 to 200 M Trolox (calibration curve) or sample was then added and the chemiluminescence was measured during 60 s. Both the Trolox and samples reduced the chemiluminescence. The rapid reduction in luminol intensity is considered as a measure of the TAR capacity calculated as nmol Trolox/mg protein. Protein concentrations were determined by Biuret method (Labtest Kit^®^) using bovine serum albumin as standard.

### 3.8. Animals

Female Wistar rats (3–4 months old, weighing 200–250 g) were employed in the experiments. The animals were kept on shelves with ventilated cages which provided 60 air cycles per hour, relative humidity ranging between 55–65%, a 12 h light-dark cycle and temperature of 22 ± 2 °C with free access to food and water. The animals were maintained in accordance with the Guiding Principles in the Care and Use of Animals of the Council of the American Physiological Society. The experimental protocol was approved by the Ethic Research Committee of PUCRS (CEUA 08/0010).

### 3.9. Carrageenan-induced Pleurisy

Animals were divided into groups of seven rats each. In the animals of the treated group, *B. trimera* was injected intraperitoneally (i.p.) in bolus (acute therapy), 30 min before the carrageenan-induced pleurisy [13]. The groups using *B. trimera* extracts were: DCM, EA, BU and AQ at doses of 25, 50 and 75 mg/kg; SAP at doses of 15, 22.5 and 30 mg/kg; and PHE at doses of 5, 10 and 15 mg/kg. AQ extract was diluted in saline while all other extracts were solubilized with 1.0% of dimethyl sulfoxide (DMSO) in saline. In another group, dexamethasone was injected (s.c.) in bolus (1.0 mg/kg), 30 min before the carrageenan-induced pleurisy. In the carrageenan group, saline solution was injected i.p. 30 min before the intrapleural (i.pl.) injection of carrageenan. In the control group, saline solution was injected both at i.pl. and i.p. routes. The pleurisy was induced by the injection of 0.2 mL of sterile saline solution (NaCl 0.9%) containing carrageenan (1%) into the right pleural space of animals under anesthesia. Animals were sacrificed 4 h later in an atmosphere of CO_2_. The chest was then carefully opened and the pleural cavity was rinsed with 2 mL of saline solution containing 1% EDTA. The exudates and rinse solution were removed by aspiration and the total volume was measured. Exudates contaminated with blood were discarded. The amount of exudates was calculated by subtracting the volume injected (2 mL) from the total volume recovered. Total leukocytes were diluted in Thoma solution (1:20) and counted in a Neubauer chamber using light microscopy. Cytological slide smears stained with May-Grünwald/Giemsa were used for differential leukocyte counts in a light microscope. The protein levels were determined by colorimetric using Biuret technique and the equipment COBAS MIRA PLUS (Roche Diagnostics). The amount of NO in the exudate was analyzed using the Griess reaction method that measures nitrite [21]. The inflammatory parameters evaluated were: volume of exudation in the pleural cavity, protein concentration in the exudate, total leukocytes count, leukocytes differential cell count and NO concentration.

### 3.10. Data Analysis

Results are expressed as means ± S.D. and statistical significance was determined by One-Way Analysis of Variance (ANOVA, *p* < 0.05).

## 4. Conclusions

In conclusion, phenolic extract of *B. trimera* presented anti-inflammatory activity at the dose of 15 mg/kg, which is very potent. Also, this latter extract presented the highest phenolic content. These values are very significant in comparison to other compounds, such as ascorbic acid. These results indicate that it was possible to obtain an extract enriched in active compounds which should contribute to the discovery of lead compounds to design new drugs.
